# The effects of Workplace violence on healthcare workers in Tunisia

**DOI:** 10.1192/j.eurpsy.2022.1794

**Published:** 2022-09-01

**Authors:** M. Mnif, N. Smaoui, R. Feki, I. Gassara, S. Omri, M. Maalej, N. Charfi, J. Ben Thabet, L. Zouari, M. Maalej

**Affiliations:** Hedi Chaker University Hospital, Psychiatry C, Sfax, Tunisia

**Keywords:** healthcare workers, violence

## Abstract

**Introduction:**

Exposure to violence affects employees and has implications for the quality of care provided.

**Objectives:**

This study aims to describe the effects of workplace violence on nurses in psychiatric and emergency departments.

**Methods:**

This was a cross-sectional and descriptive study involving 60 nurses practising in the psychiatry and emergency services at the Hedi Chaker and Habib Borguiba University Hospital in Sfax. We collected the socio-demographic and professional data of the participants using a pre-established questionnaire.

**Results:**

The average age was 35 years and 51 % of respondents were female. Ninety-three percent of the respondents were victims of an act of violence. The violence was verbal in 90%, physical in 70%, psychological in 62% and sexual in 11% of cases. The classification of acts of violence according to the scale of seriousness of the national observatory of violence in health care revealed a predominance of level 1 violence characterised by insults (66%) and level 2 violence with threats to physical integrity (65%). Level 3 violence (physical violence) was the most frequent (70%). Two cases of level 4 violence with knives were reported. These acts of violence generated wounds in 21%, fractures in 10%, haematomas in 10% and bruises in 8% of cases. Thirty-six nurses (60%) reported that the act of violence was responsible for a feeling of insecurity.

**Conclusions:**

The results of this study indicate the need for hospital center managers to set up organizational policies against workplace violence and to apply them in a rigorous and transparent manner.

**Disclosure:**

No significant relationships.

